# Unraveling resistance mechanisms in anti-CD19 chimeric antigen receptor-T therapy for B-ALL: a novel in vitro model and insights into target antigen dynamics

**DOI:** 10.1186/s12967-024-05254-z

**Published:** 2024-05-21

**Authors:** Hongzhe Li, Yuwen Wang, Rongrong Liu, Xiaoxiao Li, Ping Zhang, Ping Chen, Ning Zhao, Bing Li, Jie Wang, Yongmin Tang

**Affiliations:** 1 Department/Center of Hematology-oncology, Children’s Hospital, Zhejiang University School of Medicine, National Clinical Research Center for Child Health, Hangzhou, Zhejiang China; 2 Pediatric Leukemia Diagnostic and Therapeutic Technology Research Center of Zhejiang Province, National Clinical Research Center for Child Health, Hangzhou, Zhejiang China; 3 Department of Pediatrics, The First People’s Hospital of Wenling, Wenling, Zhejiang China

**Keywords:** CD19, CAR-T cell therapy, B-ALL, Antigen negative relapse, Resistant mechanisms

## Abstract

**Background:**

Cellular immunotherapy, represented by the chimeric antigen receptor T cell (CAR-T), has exhibited high response rates, durable remission, and safety in vitro and in clinical trials. Unfortunately, anti-CD19 CAR-T (CART-19) treatment alone is prone to relapse and has a particularly poor prognosis in relapsed/refractory (r/r) B-ALL patients. To date, addressing or reducing relapse remains one of the research priorities to achieve broad clinical application.

**Methods:**

We manufactured second generation CART-19 cells and validated their efficacy and safety in vitro and in vivo. Through co-culture of Nalm-6 cells with short-term cultured CART-19 cells, CD19-negative Nalm-6 cells were detected by flow cytometry, and further investigation of the relapsed cells and their resistance mechanisms was evaluated in vitro.

**Results:**

In this study, we demonstrated that CART-19 cells had enhanced and specific antileukemic activities, and the survival of B-ALL mouse models after CART-19 treatment was significantly prolonged. We then shortened the culture time and applied the serum-free culture to expand CAR-T cells, followed by co-culturing CART-19 cells with Nalm-6 cells. Surprisingly, we observed the proliferation of CD19-negative Nalm-6 cells around 28 days. Identification of potential resistance mechanisms showed that the relapsed cells express truncated CD19 proteins with decreased levels and, more importantly, CAR expression was detected on the relapsed cell surface, which may ultimately keep them antigen-negative. Furthermore, it was validated that CART-22 and tandem CART-22/19 cells could effectively kill the relapsed cells, but neither could completely eradicate them.

**Conclusions:**

We successfully generated CART-19 cells and obtained a CD19-negative refractory relapsed B-ALL cell line, providing new insights into the underlying mechanisms of resistance and a new in vitro model for the treatment of r/r B-ALL patients with low antigen density.

**Supplementary Information:**

The online version contains supplementary material available at 10.1186/s12967-024-05254-z.

## Background

Acute lymphoblastic leukemia (ALL) is the most common hematological malignancy in children, including B lineage (B-ALL) and T lineage (T-ALL) according to different immunophenotypes [1, 2]. With the improvement of diagnostic classification technology and the continuous optimization and application of combined chemotherapy, the 5-year overall survival (OS) rate has reached more than 90% in children [3]. However, relapsed/refractory B-ALL (r/r B-ALL) remains an important reason for the failure of treatment in pediatric leukemia [4]. Although allogeneic hematopoietic stem cell transplantation (allo-HSCT) can improve OS and disease-free survival, and reduce relapse in the treatment of leukemia patients, most patients who have undergone chemotherapy are still facing the challenge of allo-HSCT treatment due to the side effects such as severe organ damage, severe graft-versus-host disease (GVHD), as well as relapse etc. Post-transplantation-related complications are significant factors that affect the survival and the quality of life of patients.

Tumor immunotherapy, represented by chimeric antigen receptor T cell (CAR-T), is a novel approach for treating hematological malignancies. CAR-T cell therapy has exhibited high response rates, durable remission and safety in both in vitro and clinical trials, showing its huge potential for application and development. Particularly, anti-CD19 CAR-T (CART-19) cell therapy has achieved encouraging clinical effects in the treatment of r/r B-ALL cases [5–7]. Nevertheless, clinical evidence indicates that a large proportion of patients with B-ALL are prone to relapse after CART-19 cell therapy alone, and it has relatively large side effects in r/r B-ALL patients [8–10]. Tumor cells, CAR-T cells, and the tumor microenvironment play crucial roles in the relapse of malignancies, leading to poor outcomes. Optimization and breakthroughs are needed to improve antigen recognition, boost CAR-T cell function, and create combination treatment approaches.

In order to explore strategies to improve CAR-T cell efficacy, it is important to understand how tumor cells resist CAR-T cells and subsequently relapse. To date, the most characteristic mechanism of resistance is the loss of the target antigen (Ag). Several mechanisms have been reported for Ag-negative relapses, including pre-existing Ag-negative tumor cells, gene mutations or alternative splicing, alterations that affect the maturation of target Ag expression, epitope masking, and lineage switch [11, 12]. In order to prevent relapse after CAR-T cell therapy, several potential strategies, including research on new CAR targets, sequential therapy, dual/multi-target CARs, combining multiple chemotherapy and immunotherapy, as well as a variety of improved methods such as allo-HSCT, could lead to better disease remission and greater survival benefits in r/r B-ALL patients [11, 13]. Despite these issues, CAR-T cell therapy is a novel approach, and there is a need for further investigation into the emerging challenge of negative relapse and the mechanisms of antigen escape. Most of the current findings on the mechanism of negative relapse are derived from patients in clinical trials. Due to the heterogeneity of clinical samples and the inability to culture them in vitro for a long time, tumor cell lines can provide a more controlled and consistent environment for research and are considered suitable models for studying intracellular regulation and mechanisms of drug action.

In this study, we confirmed the function and efficacy of CART-19 cells cultured by regular methods in both in vitro and in vivo studies. However, we observed the emergence of CD19-negative Nalm-6 clones after antigen-specific killing of Nalm-6 with low effector: target (E: T) ratios by CART-19 cells expanded under serum-free culture conditions. The relapsed clones maintained the CD19-negative phenotype in long-term culture, generating a CD19-negative relapsed B-ALL cell line. We further observed that relapses express few and smaller CD19 proteins, and we subsequently ruled out other possible factors contributing to Ag-negativity, including pre-existing CD19^−^ Nalm-6 cells, structural changes in the B cell receptor complex, CD19 splicing variants, and promoter DNA hypermethylation. Unexpectedly, we found that these relapsed cells were transduced with the lentiviral vector during the co-culture process for the cytotoxicity assay. Additionally, we evaluated the improved anti-relapsed B-ALL activity of CD22-targeted and dual-target CD19/CD22 CAR-T cells, but were unable to eradicate relapses. Overall, our work suggests that utilizing this CD19-negative relapsed B-ALL cell line as a cellular model to explore new treatment strategies offers unique advantages and great practical significance.

## Materials and methods

### Construction of lentiviral vectors

As reported previously [14–17], we constructed CD19-BBζ-CAR, CD22-BBζ-CAR and dual-target CD22/19-BBζ-CAR comprising the anti-CD19 FMC63 single chain variable fragment (scFv) or/and anti-CD22 m971 scFv, followed by human CD8 hinge and transmembrane domains, 4-1BB co-stimulation and CD3ζ activation domains. We inserted CAR fragments into a shuttle vector consisting of a pLenti6.3 lentiviral vector backbone in which the CMV promoter had been replaced by the EF-1α promoter. Lentiviruses were generated by transfecting 80% confluent human embryonic kidney cell line (HEK293T or simply 293T) cells with the lentiviral vector using Lipofectamine 3000 (Invitrogen, USA), together with the packaging constructs including gag/pol, rev and vesicular stomatitis virus G (VSV-G). 6 h later, Opti-MEM was replaced with DMEM supplemented with 2% fetal bovine serum (FBS). After 48 to 72 h post-transfection, viruses were harvested from conditioned medium and filtered through 0.45 μm filters (Millipore, USA) to remove cellular debris, then concentrated and purified with polyethylene glycol (PEG)-8000 by centrifugation.

### Cell culture

The human B-ALL Nalm-6 cell line and 293T cell line were acquired from the American Type Culture Collection (ATCC, USA). Cells were cultured at 37 °C in a humidified environment with 5% CO_2_ and identified bimonthly. B-cell surface markers of Nalm-6 cells were routinely examined. High glucose DMEM (Gibco, USA) with 10% FBS (Gibco, USA) was the standard culture medium for 293T cells. Nalm-6 cells were cultured in RPMI-1640 (Gibco, USA) supplemented with 10% FBS. Typically, T cells were cultivated in X-vivo 15 Medium (Lonza, USA) supplemented with 5% FBS and IL-2 (50U/ml, PeproTech, USA). Serum-free medium cultured T cells were cultivated in TexMACS™ Medium (Miltenyi Biotec, Germany) with addition of IL-2 (50IU/ml, Miltenyi Biotec, Germany).

### Expansion and transduction of human T cells

Venous blood samples were collected from healthy adult volunteer donors. Untouched and highly purified T cells were isolated directly from whole blood using the RosetteSep™ Human T Cell Enrichment Cocktail (Stemcell, Canada) and Ficoll-Paque PREMIUM (GE Healthcare, USA) following the manufacturer’s instructions. Purified T cells were activated using T Cell TransAct (Miltenyi Biotec, Germany) on day 0. After 24–36 h of activation, lentivirus was added at a multiplicity of infection (M.O.I.) of 5 to transfer T cells and removed by centrifugation on day 3. Subsequently, cells were split and the culture medium was exchanged with fresh medium and IL-2 every 2–3 days. Daily samples were taken and counted, and cell density was calculated based on absolute counts. If the short-term culture was performed, T cells were purified from whole blood and activated as described above, and expanded in TexMACS Medium (Miltenyi Biotec, Germany) supplemented with IL-2 (50 IU/ml, Miltenyi Biotec, Germany) with or without FBS (5%). Lentivirus was added at a M.O.I. = 5 after 24 h of T cell activation. 72 h after transduction, CAR-T cells were collected by centrifugation and washed with PBS, followed by anti-tumor activity assays.

### The anti-tumor activity of short-term cultured CART-19 cells

The short-term, serum-free cultured CART-19 cells were co-cultured with Nalm-6 cells in TexMACS Medium supplemented with IL-2. The CART-19 cells that underwent short-term preparation in the serum-containing medium were co-cultured with Nalm-6 cells in RPMI-1640 supplemented with IL-2 and 5% FBS. CART-19 and untransduced T effector cells were co-cultured with target cells at E: T ratios of 1:5, 1:1, and 5:1 in a total volume of 1 ml (1 × 10^6^ cells) in the 24-well plates. Every two days, the culture supernatants were pipetted, and fresh culture medium was added. Co-cultured cells were analyzed using flow cytometry, and cell images were captured with Olympus CKX53 microscopy (Olympus, Japan).

### Cytotoxicity LDH assay

Lactate dehydrogenase (LDH) release assays were performed according to manufacturer’s instructions using a Cytotoxicity LDH Assay Kit (Dojindo, Japan). Briefly, the indicated CAR-T and untransduced T cells were co-cultured with target cells at E: T ratios of 0.2:1, 1:1, and 5:1 in a total volume of 100 µL (3 × 10^5^ cells) in the wells of 96-well plates at 37℃ for 6 h, 18 h and 24 h. Supernatants were collected, and LDH was measured using a colorimetric reaction (absorbance at 490 nm). As part of the measurements, we measured spontaneous and maximum release of effector and target cells, and the percentage of specific cell lysis was calculated using a standard formula: Cytotoxicity (%) = (experimental release - spontaneous release) / (maximal release - spontaneous release) × 100.

### Flow cytometry (FCM) analyses

To evaluate CAR expression after 7–10 days of culture, CART-19 cells were washed once and incubated with goat anti-human biotin conjugated anti-Fab antibody (Jackson ImmunoResearch, USA) for 30 min at room temperature. The cells were then washed twice and stained with phycoerythrin (PE) streptavidin (BD bioscience, USA) for 15 min. CART-22 cells and CART-22/19 cells were washed once and incubated with CD22 Fc Alexa Fluor® 647 Protein (R&D Systems, USA) for 15 min.

To detect in vitro cytotoxicity of CART-19 cells, transduced and untransduced T cells were co-cultured with Nalm-6 cells (total 1 × 10^6^ cells) at E: T ratios (0.2:1, 0.5:1, 1:1, 5:1) for 6, 24 and 72 h. Cells were pipetted to incubate with antibodies for 30 min at room temperature in the dark and washed twice with PBS. Then cell pellet was resuspended in cell staining buffer (BioLegend, USA) and incubated with 7-amino-actinomycin D (7-AAD) for 5 min in the dark. The flow cytometric analysis of the cells was performed immediately after the incubation with 7-AAD (BioLegend, USA). Cytotoxicity (%) was calculated as the proportion of CD19^+^7-AAD^+^ cells to all cells.

The following antibodies (clones) were used for phenotypical analysis of cells: CD45 (HI30), CD45RA (L48), CD45RO (UCHL1), CD62L (DREG-56), CD3 (SK7), CD4 (RPA-T4), CD8 (RPA-T8), CD10 (HI10a), CD19 (SJ25C1/HIB19), CD20 (2H7), CD22 (HIB22), CD81 (JS-81), CD58 (1C3), and all were from BD Bioscience. Cells were pipetted to incubate with antibodies for 30 min at room temperature in the dark and then washed twice with PBS. For staining of cytoplastic CD19 (cyCD19), the cells were permeabilized using permeabilizing solution 2 (BD, USA) prior to intracellular immunofluorescence staining with the CD19 antibody (clone: D4V4B, Cell Signaling Technology, USA) and secondary antibody FITC conjugated goat anti-rabbit IgG H&L (Abcam, USA). In animal experiments, 50 µL of peripheral blood or 10^6^ individual tissue cells were incubated with antibodies for 30 min at room temperature in the dark, followed by incubation in red blood cell lysis buffer and PBS washing.

Data from these experiments were collected using a BD FACSCanto II flow cytometer and BD FACS Diva software, and analyzed using FlowJo version 10 (Treestar, USA).

### LiquiChip

Cytokines and chemokines in the culture supernatant were quantified utilizing a Bio-Plex pro human cytokine 48-plex screening panel kit (Millipore, USA) in accordance with the manufacturer’s guidelines and detected by the Bio-Plex 200 System (Bio-Rad, USA). Advanced heatmap plots were generated using the OmicStudio tools available at https://www.omicstudio.cn.

### Mouse models and CART-19 treatment

The hemostatic effects of the CART-19 cells were evaluated in vivo using animal models. The 6- to 8-week old NOD.Cg*-Prkdc*^*scid*^*Il2rg*^*tm1Wjl*^/SzJ (NSG) mice were purchased from Jiangsu Jicui Yaokang Biotechnology Co., Ltd (Jiangsu, China; Animal Certificate No: SCXK (SU) 2023-0009), housed and treated under specific pathogen-free conditions at the Experimental Animal Facility of Hangzhou Medical College. All animal experiments were carried out according to the applicable guidelines and regulations approved by the Hangzhou Medical College. B-ALL mouse models were established by the tail vein injection of 3 × 10^5^ Nalm-6 cells, and then randomly divided into 3 groups (*n* = 8). On the following day, they were randomized to receive one of the following tail vein injections: (1) PBS, (2) 6 × 10^5^ untransduced mock T cells in PBS, or (3) 6 × 10^5^ CART-19 cells in PBS. One more group added to receive PBS (*n* = 4). Every two to three days, mice were checked for changes in body weight. Orbital blood collection was performed weekly after administration. Every week, the dynamics of tumor cells and T cells from peripheral blood were detected by FCM. Death, loss of more than 20% of body weight from baseline, or the presence of specific signs (e.g., hindlimb weakness or paralysis) were defined as survival endpoints.

### H&E staining

Fresh thigh bones of mice were sampled and put into pre-prepared 10% formalin for fixation, followed by decalcification in ethylene diamine tetra-acetic acid (EDTA) for one month. After that, the tissues were dehydrated with a gradient of alcohol from low to high concentrations and then placed in xylene for transparency. Subsequently, the tissues were embedded by dipping them into melted paraffin wax and waiting for the paraffin wax to be completely immersed in the tissues. The embedded tissues were cut into thin slices, typically 5–8 μm thick, and then attached to slides. Finally, the paraffin was removed from the sections with xylene, and after hematoxylin-eosin (H&E) staining and dehydration, the sections were sealed for observation. All slices were scanned and analyzed by a Pannoramic MIDI scanner (3DHISTECH, Hungary).

### Cloning CD19 CDS and sequencing

RNeasy kit (QIAGEN, Germany) was utilized to extract total RNA from wild-type (WT) and relapsed Nalm-6 cells. Then the PrimeScript RT reagent kit was used to do reverse transcription with 1µg of RNA, following the manufacturer’s instructions (Takara, Japan). The coding sequence (CDS) of *CD19* gene was amplified from cDNA using the following primer pair, 16F (5’–GAGAGTCTGACCACCATGCC-3’) and 1894R (5’– GGAATACAAAGGGGACTGGAAG-3’). PCR reaction system was as follows: 5× GXL buffer 10 µL, dNTP Mixture (2.5 mM) 4 µL, forward primer (10 µmol/L) 1 µL, reverse primer (10 µmol/L) 1 µL, cDNA 2 µL, PrimeSTAR GXL DNA Polymerase 1 µL, ddH_2_O was supplemented to 50 µL. PCR was performed 30 cycles of 98 °C for 10 s, 60 °C for 15 s, 68 °C for 2 min, and a final 72 °C for 5 min. Then, amplification products were detected by 1% agarose gel electrophoresis and purified by the QIAquick gel extraction kit (QIAGEN, Germany). Interested fragments were ligated into the pMD20-T vector using the mighty TA cloning kit (Takara, Japan) and then transformed into DH5α competent cells. Subsequently, 10–100 µL from each transformation were plated on LB agar plates (containing X-Gal, IPTG and Ampicillin). White colonies were chosen and cultured for plasmid isolation, and positive recombinants were analyzed by restriction analysis and Sanger sequencing.

### RNA sequencing and differential expression genes (DEGs) analysis

According to the manufacturer’s instructions, total RNA was isolated and purified using TRIzol reagent (Thermo Fisher, USA). The amount and purity of the total RNA were assessed by NanoDrop ND-1000 (NanoDrop, USA). The integrity of the RNA was then examined by Bioanalyzer 2100 (Agilent, USA) and verified by agarose electrophoresis. Qualified RNA samples (> 1 µg, concentration > 50 ng/µL, RNA integrity number > 7.0) were used for sequencing library construction. Poly (A) RNA was specifically captured using Dynabeads Oligo (Thermo Fisher, USA) and fragmented at 94 °C. The fragmented RNA was synthesized into cDNA by reverse transcription. cDNA was then converted into U-labeled second-stranded DNAs using *E. coli* DNA polymerase I, RNase H (NEB, USA) and dUTP Solution (Thermo Fisher, USA), and screened and purified for fragment size using magnetic beads. DNAs was digested with UDG enzyme (NEB, USA) and PCR was performed to form a sequencing library with average fragment size of 300 ± 50 bp. Finally, the illumina Novaseq™ 6000 (LC Bio Technology CO., Ltd., China) was used for the 2 × 150 bp paired-end sequencing (PE150) according to standard operating procedures provided by the manufacturer. Following quality control and filtering, clean reads were mapped to the genome (Homo sapiens, GRCh38) using HISAT2 package. Gene abundance was then estimated using StringTie and ballgown, with FPKM (fragment per kilobase of transcript per million mapped reads) values calculated. DEGs analysis was conducted using the DESeq2 software to compare the normal group with the experimental group. Genes meeting the criteria of a false discovery rate (FDR) below 0.05 and an absolute fold change of at least 2 were identified as differentially expressed genes.

### Real-time quantitative PCR (qRT-PCR)

cDNAs were prepared the same way as preparing T-A clone samples as described above. Genomic DNA was isolated from 2 × 10^6^ cells from WT and relapsed Nalm-6 cells using the TIANamp genomic DNA kit (TIANGEN, China) following the manufacturer’s protocol. Primers used for different regions of the CD19 mature mRNA as previously described [18], and PCR primers specific to the FMC63, CD3ζ, 4-1BB [15] and VSV-G [19] fusion gene in Nalm-6 cells are listed in Additional file 1 (Table 1). qRT-PCR was performed using TB Green Premix Ex Taq or Probe qPCR Mix (TaKaRa, Japan) according to the operating protocol provided by the manufacturer. The levels of gene expression were normalized to *ACTB*. The reactions were carried out on the StepOnePlus real-time PCR system from Applied Biosystems (Thermo Fisher, USA).

### Western blot

Cells were washed with PBS, collected by centrifugation, and fully lysed by adding cell lysis buffer (containing 1 mM PMSF) until no obvious cell precipitation was observed. Protein concentration was determined using the BCA Protein Quantification Kit (Yeasen, China). 10 µg of protein sample was taken and added to loading buffer, then denatured in boiling water for 10 min, followed by SDS-PAGE electrophoresis to separate the proteins. The proteins in the SDS-PAGE gel were transferred to a PVDF membrane, and the transfer time was adjusted based on the size of the proteins. The membrane was washed with TBS and then blocked in 5% skimmed milk for 1 h. Subsequently, membranes were incubated with primary CD19 antibodies (clone D4V4B, Cell Signaling Technology, USA; clone OTI5F3, Origene, USA) at a dilution of 1:1000 at 4 °C overnight and then washed 3 times and incubated with HRP-conjugated secondary antibodies (GAR007 or GAM007, MultiSciences, China) at a dilution of 1:5000 for 2 h at room temperature. Proteins were detected using an ECL Detection Reagent (Thermo Fisher, USA) with the Amersham Imager 600 spectrum (GE HealthCare, USA).

### Immunofluorescence analysis

For CD19 staining, the cells were washed with PBS through centrifugation. The cells were fixed with 4% paraformaldehyde for 15 min and permeabilized with saponin (Solarbio, China) for 10 min at room temperature. Subsequently, the cells were subjected to a 1-hour blocking step using 5% BSA and then labeled with CD19 (Intracellular Domain) (D4V4B) XP® Rabbit mAb (Cell Signaling Technology, USA) at 4 ℃ overnight. After washed with PBS for three times, cells were incubated with secondary antibody CY3 conjugated anti-rabbit IgG (Abcam, USA) for 1 h and then stained with Alexa Fluor 488 Mouse Anti-Human CD19 (BD bioscience, USA) for 30 min. Finally, mounting medium with DAPI (Abcam, USA) was added for nuclei staining. The images were collected by Leica DMi8 microscopy (Leica, Germany).

To identify the presence of anti-CD19 CAR on the surface of Nalm-6 cells, the cells were treated with 4% paraformaldehyde for 20 min for fixation, followed by a 1-hour incubation in 5% bovine serum albumin (BSA) for blocking. Then, the cells were incubated and stained with Alexa Flour 647-conjugated Goat Anti-Mouse IgG antibody (Jackson ImmunoResearch, USA) for 1 h followed by Alexa Fluor 488 Mouse Anti-Human CD19 for 30 min. Finally, the cells were counterstained with DAPI and used for confocal images on a Leica TCS SP8 Laser Scanning confocal microscope (Leica Microsystems, Germany).

### Lentiviral integration site analysis

Genomic DNA samples were extracted the same way as preparing PCR samples as described above and subjected to quality control before use. The frequency and location of CAR19 integration into genomic loci were assessed and analyzed using established methodologies [20, 21]. Briefly, genomic DNA was fragmented by ultrasonic interruption, followed by end repair, A-addition and linker ligation using the VAHTS universal DNA Library Prep Kit (Vazyme, China). Primers were designed according to lentiviral LTR sequences and asymmetric junction sequences, and lentiviral integration sites were enriched by the nested linker-mediated PCR (LM–PCR) and ligated with illumina adapter sequences. Sequencing of the pooled library was performed on the Illumina MiSeq platform. After preprocessing and quality control, clean reads were aligned to the human reference genome using Bowtie2. Based on the gene databases and ChIPseeker package, the gene information, gene function regions, distance to transcription start sites (TSS) and CpG islands, and regions of the proto-oncogene/oncogene were annotated and counted at the integration site.

### Statistics

Statistical calculations were performed using GraphPad Prism 9.4. Data are presented as the mean ± standard deviation (s.d.) or standard error of the mean (s.e.m.). Differences between groups were analyzed by two-tailed Student’s t tests. Kaplan-Meier curves were constructed for survival analysis. *p < 0.05, **p < 0.01, and ***p < 0.001 were considered to be statistically significant.

## Results

### Efficient generation and primary functional analysis of CART-19 cells

**Fig. 1:**
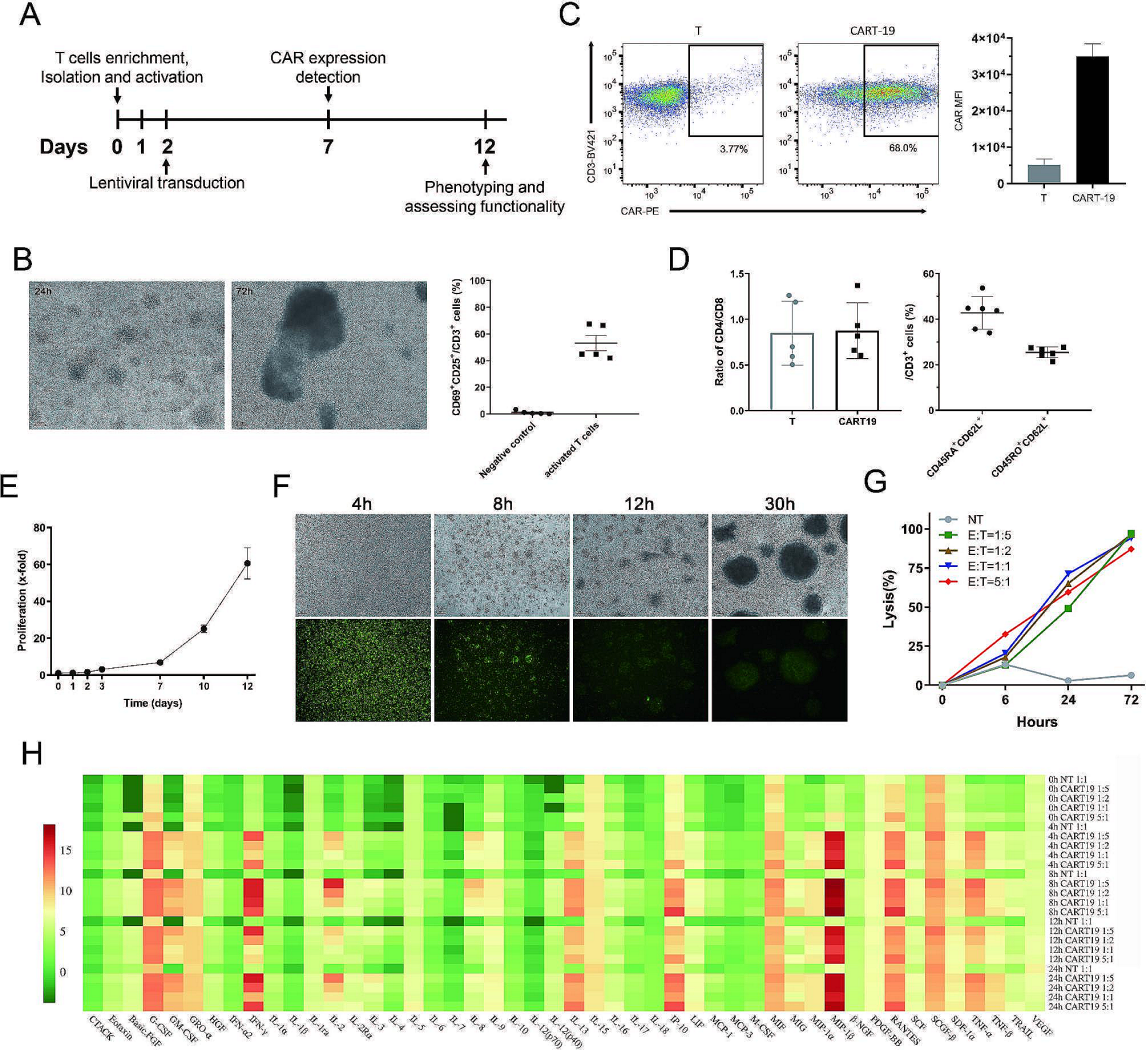
Proliferation and specific cytotoxic effects of CART-19 cells. **A** The design of the CAR-T cell construction experiments. **B** Morphological images of activated T cells clustered after 24 h and 72 h of incubation with TransAct CD3/28 beads. **C** Flow cytometric analysis of CAR expression on the surface of mock T, and CART-19 cells with biotin-conjugated anti-Fab antibody followed by PE-conjugated streptavidin. Gating was based on the same cells stained with isotype-matched antibody. The median fluorescence intensity (MFI) was calculated for CAR-T population in the PE fluorescence channel (right column). This result is the representative of three separate experiments using cells from healthy volunteer donors. **D** The phenotypic characterization of CART-19 cells by flow cytometry. The ratio of CD4^+^ / CD8^+^ T cells (left) and the proportion of T_N/CM_ (right) are shown. **E** Growth curves of CAR-T cells. Data represent the mean ± s.d. of three separate experiments. **F** Cytolytic activities of CART-19 cells in cell assays. Nalm-6 cells were labeled with CFSE labeling reagent (Sigma-Aldrich, USA) and co-cultured with CART-19 cells at the E: T ratio of 1:1 for 30 h. The presence of CFSE-labeled cells was observed by microscopy. Bar, 100 μm. **G** Cytotoxic activity of mock NT and CART cells against Nalm-6 cells. The effector cells were co-cultured with target cells at E: T ratios of 1:5, 1:2, 1:1 and 5:1 with a total cell number of 1 × 10^6^. **H** Dynamic changes of cytokine secretion profile of CART-19 cells during 24 h after co-culture with Nalm-6 cells at E: T ratios of 1:5 to 5:1. Data were visualized by heatmap. Concentrations (pg/ml) of cytokines and chemokines in the supernatant were detected by multiplex immunoassay and the values were log2 transformed

Successful gene therapies with CAR-expressing T cells rely on the efficiency of CAR transduction and the quality of CAR-T cells. We have optimized methods for culturing and transducing T cells to produce potent CART-19 cells from the blood of healthy adults. The aggregation of activated cells into clumps could be observed, and 53% of CD25^+^CD69^+^ T cells were obtained after incubation with TransAct CD3/28 beads for 40 h (Fig. 1B). By using lentiviral vectors and transducing at a multiplicity of infection (M.O.I.) of 5, we achieved a high level (∼ 68%) of CD19-BBζ CAR expression in primary human T cells, and the mean fluorescence intensity (MFI) of CAR staining in the transduced T cells was much higher than in untransduced T cells (Fig. 1C). In order to demonstrate the proliferative potential and anti-tumor activity of less differentiated CAR-T cells generated, we detected the phenotypes of CART-19 cells after 10 ∼ 12 days of culture. It was found that the ratio of CD4/CD8 cells was approximately 0.88, and the proportion of central memory T cells (Tcm, CD62L^+^CD45RO^+^) in CAR-T cells was about 25.4%, while the proportion of naive T cells (Tn, CD45RA^+^CD62L^+^) was about 42.8% (Fig. 1D). Using our established low-serum T cell culture system, CAR-T cells could be expanded more than 50-fold in about 12 days in vitro (Fig. 1E). Then, CART-19 cells were co-cultured with Nalm-6 cells at E: T ratios ranging from 1:5 to 5:1 to assess the cytotoxicity of CART-19. For observing cell killing activity at a 1:1 E: T ratio, the data suggested that the cytolytic function of the CART-19 cells exhibited robust and rapid activity (Fig. 1F). Notably, after co-culturing at an E: T ratio of 1:5 for 24 h, the CART-19 cells demonstrated significantly greater efficacy in eliminating tumor cells compared to the NT (or T, non-transduced T) cells, which were co-cultured with Nalm-6 cells at an E: T ratio of 1:1 (Fig. 1G). Finally, we analyzed the concentrations of cytokines and chemokines in the supernatants of CART-19 cells co-cultured with Nalm-6 cells. With tumor cell stimulation, the CART-19 cells exhibited heightened functional cytokine and chemokine secretion, especially the production of GM-CSF, IFN-γ, IL-2, IL-4, IL-8, IL-12, IL-13, MIP-1α/β, and TNF-α/β (Fig. 1H).

### Evaluating the antitumor activity of CART-19 cells in vivo

**Fig. 2:**
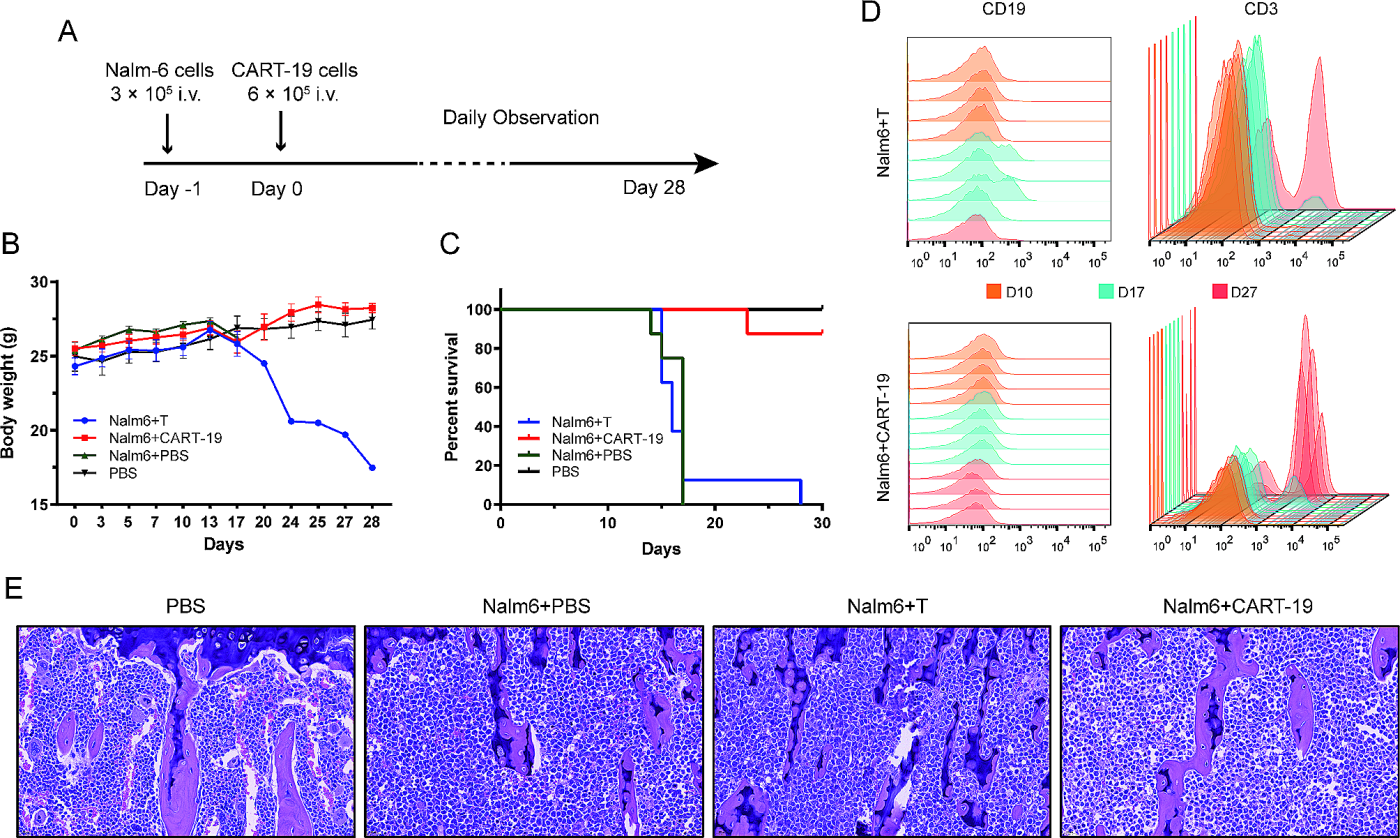
In vivo functional evaluation of CART-19 cells. **A** Model system and experimental regimen. NSG mice were injected with PBS (PBS group, *n* = 4) or Nalm-6 cells on day − 1. One day after tumor implantation, mice were randomized to receive PBS (Nalm6 + PBS group, *n* = 8), untreated mock T cells (Nalm6 + T group, *n* = 8), or CART-19 cells (Nalm6 + CART-19 group, *n* = 8). Animals were monitored daily after tail vein injection. **B, C** Body weight (**B**) and survival curves (**C**) of mice from (**A**). Values represent mean ± s.e.m. **D** Persistence of tumor and CAR-T cells from blood of treated mice in the Nalm6 + T and Nalm6 + CART-19 groups by flow cytometry analysis. Tumor cells were identified as CD19^+^ cells and T cells were identified as CD3^+^ cells. Representative flow cytometric data from four mice of each group are shown. **E** Representative morphological images of bone marrow from mice at the end of study. The final magnification of all images was 40×

Based on the above in vitro findings and an established Nalm-6 cell-based B-ALL model, we utilized T cells as a reference to evaluate the efficacy and anti-leukemic responses of CART-19 cells in NSG mice in vivo. Tail vein injection of Nalm-6 cells (Nalm6 + PBS group) resulted in the progressive development of leukemia in mice, with significant infiltration into the bone marrow and blood, eventually leading to the rapid death of the mice (Fig. 2C-E). The T cell-treated group exhibited a similar trend to the control group in terms of weight loss, survival time, and tumor growth rate, indicating that non-transduced T cells have little or no impact on the development of leukemia (Fig. 2B-E). The Nalm6 + CART-19 group showed significant antileukemic activity when 6 × 10^5^ CAR-T cells were injected 1 day after inoculation of 3 × 10^5^ Nalm-6 cells in mice (Fig. 2A-C). Because CART-19 cells were rapidly activated and proliferated in vivo under CD19 antigen-specific stimulation, the percentage of CD19^+^ cells in peripheral blood of the Nalm6 + T cell group (mean = 11.97, s.d.: 9.88) was substantially higher than that of the Nalm6 + CART-19 group (mean = 0.64, s.d.: 0.28) at day 17 after injection (Fig. 2D), but the difference was no statistical significance (*P* = 0.062).

### Loss of tumor surface antigen following CART-19 therapy

**Fig. 3:**
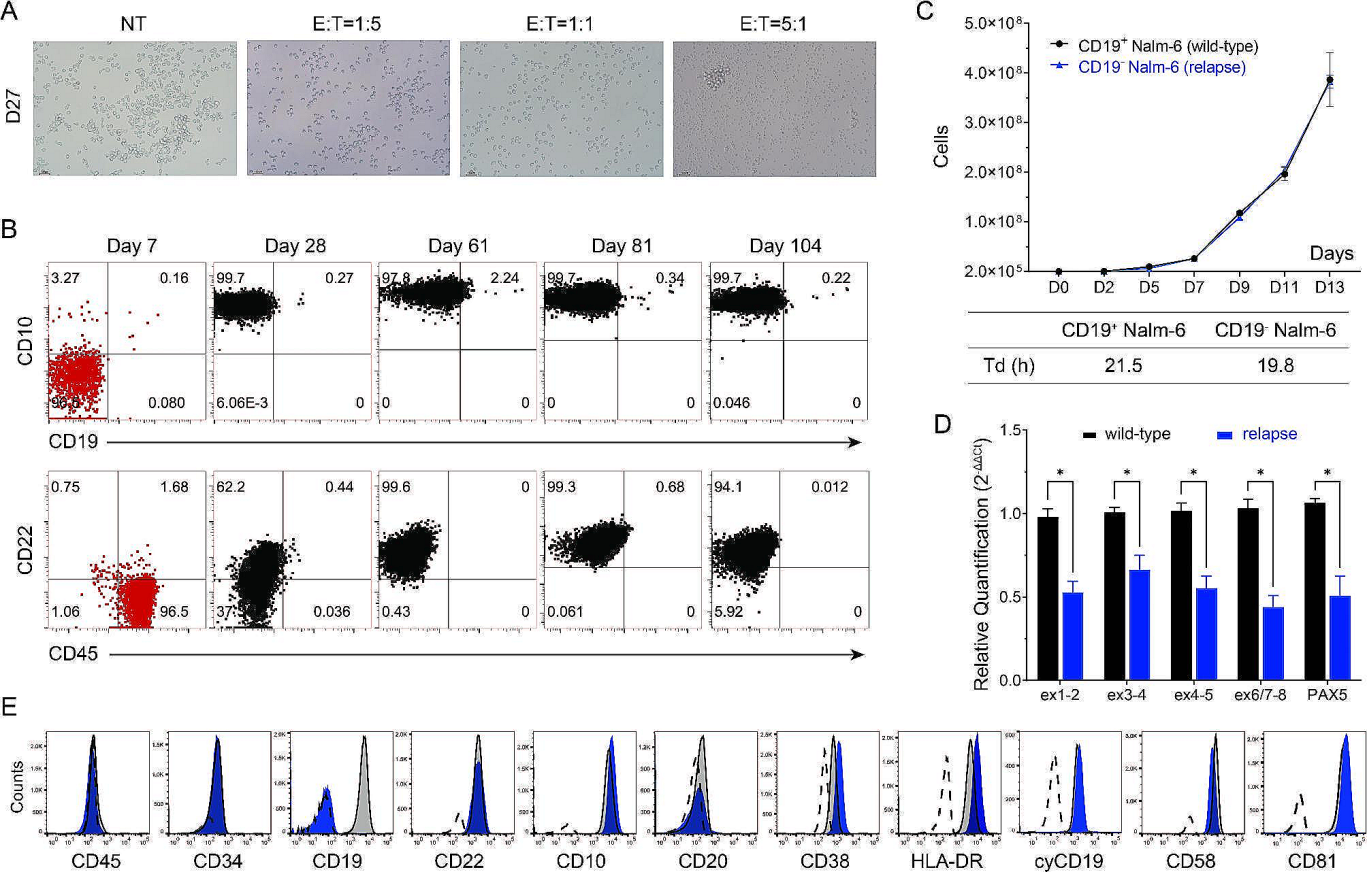
Expansion and phenotypic identification of antigen-negative relapsed cells following CART-19 treatment. **A** Morphology of CART-19 cells and Nalm-6 cells co-cultured in vitro for 27 days. The CART-19 cells were expanded in the serum-free TexMACS Medium-TransAct-IL-2 culture system. **B** Long-term immunophenotyping of co-cultured cells of CART-19 and Nalm-6 cells (E: T = 1:5) by flow cytometry. The co-culture cells were marked in red on day 7, while other days of the experiment were marked in black. **C **In vitro proliferation (line) and doubling time analysis (table) of relapsed and wild-type Nalm-6 cells. **D** qRT-PCR analysis of the expression of different regions of CD19 mRNA and PAX5 mRNA in relapsed CD19^−^ Nalm-6 samples. CD19^+^ wild-type Nalm-6 cells were applied as the control group while using *ACTB* as an internal reference, and graphs show mean ± s.e.m. of relative expression levels. *p < 0.05 indicates a significant difference. Data are representative of three independent experiments. **E** Flow cytometric profiles of immunophenotypes of relapsed CD19^−^ Nalm-6 cells (blue) compared to CD19^+^ wildtype Nalm-6 cells (grey) after 317 days of continuous culture in vitro. The results of the isotype control are shown (dashed line)

The aforementioned results indicate that we have developed an effective expansion protocol for CAR-T cells in vitro, and further verified anti-tumor activity of CART-19 cells both in vivo and in vitro. Despite encouraging results, relapse risk is associated with the ability of CAR-T cells to proliferate and their long-term persistence, while the conditions and duration of cell culture have a significant impact on CAR-T cell potency. Then, we shortened cell culture time and tested different culture systems to expand T cells, followed by co-culturing CART-19 cells expanded by the serum-free TexMACS Medium-TransAct-IL-2 culture system with Nalm-6 cells. Unexpectedly, we observed the presence of CD10^+^CD19^−^ cells around 28 days, which might be CD19-negative Nalm-6 cells due to antigen loss (Fig. 3A and B). However, no relapse of CD19-negative cells was detected after killing Nalm-6 by CART-19 cells expanded in a 5% serum TexMACS Medium-TransAct-IL2 culture system (Additional File 2: Fig. 1). Subsequently, the phenotypes of CD45, CD19, CD10, and CD22 were detected consecutively, and the results showed that the phenotype of the relapsed cells was stable and remained CD45^−^CD19^−^CD10^+^CD22^+^ (Fig. 3B). Comparative assays of WT Nalm-6 cells and relapsed Nalm-6 cells revealed consistent growth characteristics, including doubling time, cell cycle, and karyotype (Fig. 3C; Additional file 3: Fig. 2). qRT-PCR assays showed that CD19 expression in CD19^−^ Nalm-6 cells decreased by half compared to WT cells, and there were statistical differences (*P* = 0.002 ∼ 0.019, Q = 0.012 ∼ 0.022) (Fig. 3D). Meanwhile, the expression of *PAX5* decreased by 50.9% (*P* = 0.037) (Fig. 3D). After continuous subculture for 317 days, the relapsed cells maintained the CD19-negative phenotype. Other phenotypic markers, including CD45, CD34, CD10, CD20, CD22, CD38, HLA-DR, CD58, and CD81, were remained the same as WT Nalm-6 cells (Fig. 3E).

### Observation of CD19 gene transcription and expression in relapsed CD19^-^ Nalm-6 cells

**Fig. 4:**
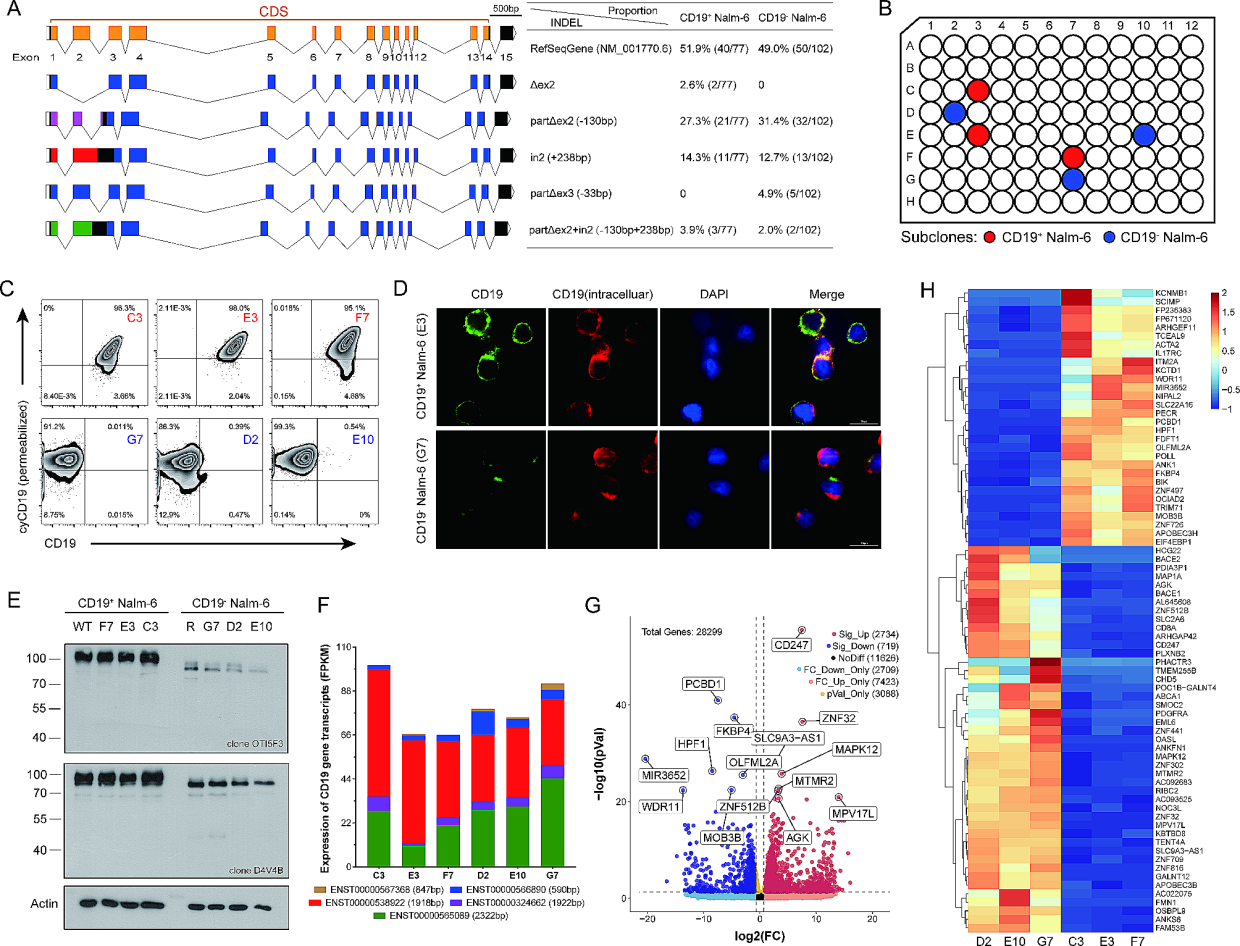
Transcription and expression of CD19 gene in the relapsed Nalm-6 cells. **A** Detection of CD19 transcripts in relapsed CD19^−^ Nalm-6 and wildtype Nalm-6 cells by TA cloning and sequencing. Sequences were compared to the reference sequence gene (NM_001770.6) and visualized by Exon-Intron Graphic Maker from WormWeb.org. Six alternatively spliced isoforms of CD19 mRNA were obtained and the percentages of them were shown separately, including the full-length (FL), skipping exon 2 (Δex2), partial deletion (partΔex2 and partΔex3), retention of intron 2 (in2) and combination of partΔex2 and in2. Of these, partΔex2, in2 and partΔex2 + in2 shift the reading frame, predicting that the expressed CD19 protein will be divided into three shortened proteins (purple, red and green) and a truncated CD19 protein (blue). **B** Schematic demonstration of single-cell purification of wild-type Nalm-6 cells (red) and relapsed CD19^−^ Nalm-6 cells (blue) by the limiting dilution method. **C** Flow cytometric profiles of membrane CD19 and cytosolic CD19 in subclones of Nalm-6 (red) and relapsed CD19^−^ Nalm-6 cells (blue). **D** Fluorescence microscopy of CD19 expression on the surface and cytoplasm using two anti-CD19 antibodies (HIB19, extracellular, green; D4V4B, intracellular, red) and DAPI (blue). **E** Immunoblotting for CD19 in protein lysates from wild-type Nalm-6 cells and relapsed CD19^−^ Nalm-6 cells using antibodies recognizing the extracellular domain (clone OTI5F3 from Origene, N-terminal) or the cytosolic domain (clone D4V4B from Cell Signaling, intracellular domain). **F** Expression of CD19 gene transcripts in RNA sequencing data from wild-type Nalm-6 and relapsed Nalm-6 subclones. **G, H** Volcano (G) and heatmap (H) plots demonstrating changes in differential gene expression of subclones measured by RNA sequencing. Differentially expressed gene (DEG) analysis identified 3453 DEGs with |log2Fc| ≥ 1 and *p* value < 0.05, and the heatmap for top 75 DEGs is shown

To further characterize the CD19 gene expression of the relapsed cells, we performed TA cloning, single-cell purification, and transcriptome analysis. RT-PCR and T-vector-based TA cloning were used for cloning the cDNA of the CD19 gene and for sequencing. We observed six alternatively spliced CD19 mRNA isoforms in Nalm-6 cells and post-CART-19 relapses, including full-length (FL), exon 2 deletion (Δex2), partial deletions (partΔex2 and partΔex3), and the retention of intron 2 (in2) (Fig. 4A). This is consistent with the CD19 isoforms observed in children with B-ALL in clinical settings (Additional file 1: Table 2). The identified alterations in exons allowed expression of N-terminally truncated CD19 variants and there was no evidence to indicate the *de novo* mutations in the relapsed Nalm-6 cells that primarily affect exon 2, as reported in other studies [18, 22, 23]. Furthermore, all splicing mutation types were subclones and collectively accounted for no more than 50% of CD19 alleles, implying the existence of additional mechanisms of gene inactivation in the relapsed cells. To confirm the continued expression of the CD19 gene in the relapsed cells, we conducted single-cell purification by the limiting dilution method to generate subclonal populations, achieving C3, E3, and F7 subclones of Nalm-6 cells and D2, G7, and E10 subclones of the relapsed cells (Fig. 4B). Although the surface marker CD19 was not detectable, intracellular domains of CD19 were identified in the relapsed subclones, and a limited quantity of the extracellular epitope of the CD19 molecule was recognized around the nucleus through immunofluorescence detection (Fig. 4C, D). However, the relapsed subclones expressed lower levels of CD19 protein, which were also smaller in size compared to the wildtypes. This was particularly evident in the N-terminal region of the full-length CD19 protein targeted by the OTI5F3 antibody (Fig. 4E). We next investigated the overall changes in the transcriptome of relapsed cells using RNA-seq. The results confirmed that CD19 transcripts were preserved in the relapsed subclones and that there was no new transcript type (Fig. 4F). Differentially expressed gene (DEG) analysis comparing relapsed subclones to WT clones identified 3453 DEGs with |log2Fc| ≥ 1 and *p* value < 0.05, and the heatmap for the top 75 DEGs was shown (Fig. 4G, H). Unexpectedly, the RNA-seq data also suggested that CD247 and CD8A genes were significantly upregulated in relapsed subclones. Considering the CAR used in this study incorporated the hinge and transmembrane regions of the CD8a molecule and the cytoplasmic portion of the CD3ζ molecule, we therefore hypothesized that the Nalm-6 cells were transduced by lentiviruses during the co-culturing process with CART-19 cells.

### Characterization of CAR-Nalm-6 cells and anti-CD22 CAR-T cell therapy as a salvage treatment

**Fig. 5:**
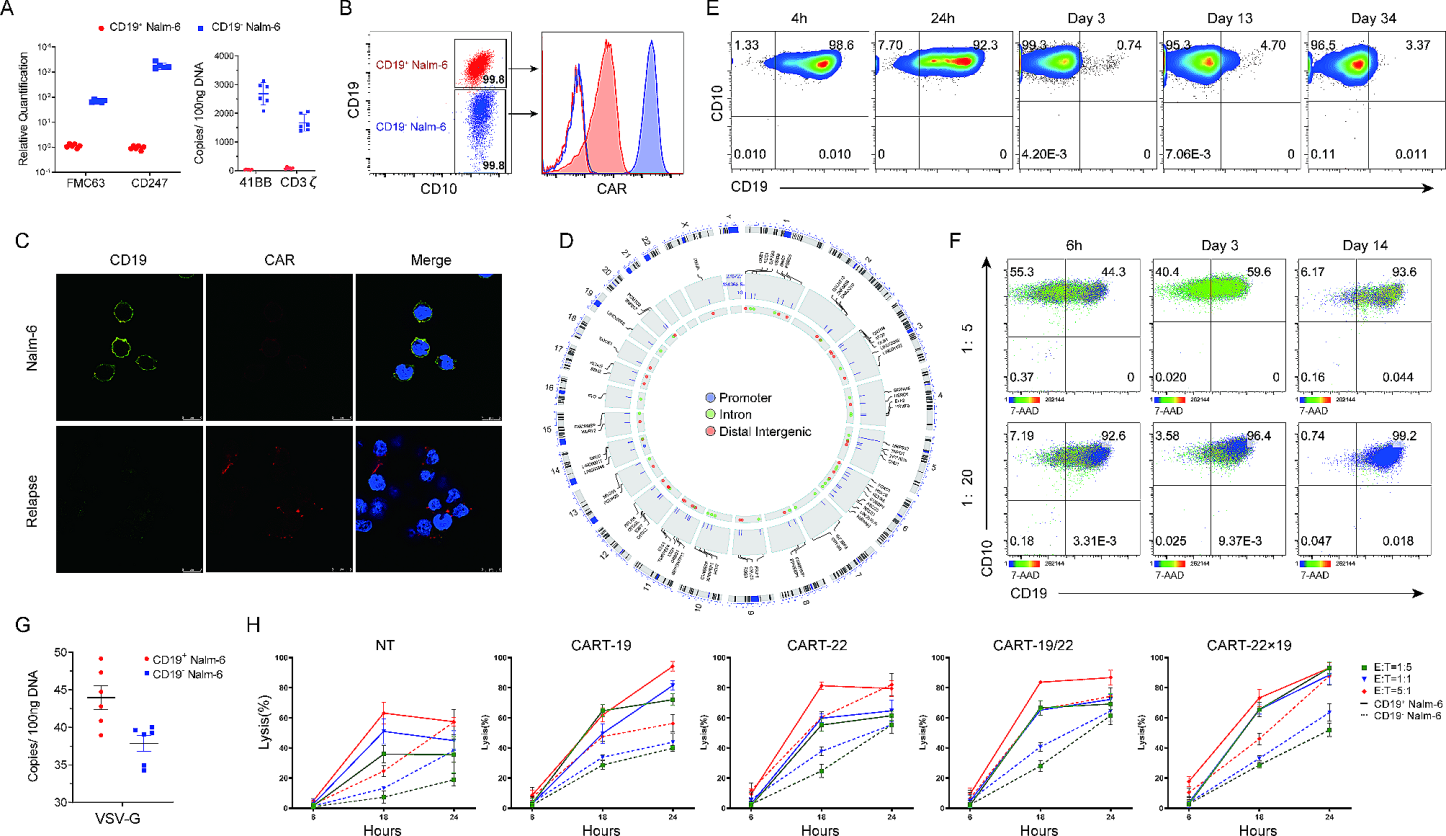
Observation of CD19-BBζ-CAR expression in relapsed Nalm-6 cells and salvage treatment. **A** Detection of FMC63 and CD247 transcripts and 4-1BB gene of CAR in CD19^+^ Nalm-6 (red) and relapsed CD19^−^ Nalm-6 cells (blue) by qRT-PCR. Data of left bar graph represent the relative quantification using ACTB as the internal reference. Error bars represent s.d. The data are the representative of three independent experiments. **B** Expression of CD19 and CAR on CD19^+^ Nalm-6 cells and relapsed CD19^−^ Nalm-6 cells analyzed by flow cytometry (representative of 3 experiments). Merge Graphs, the blue dots represent CD19^−^ Nalm-6 cells and the red dots represent Nalm-6 cells. **C** Confocal imaging of Nalm-6 cells and relapsed CD19^−^ Nalm-6 cells using Alexa Flour 488-conjugated anti-CD19 antibody (green), Alexa Flour 647-conjugated anti-CAR19 antibody (red), and DAPI (blue). **D** Lentiviral integration sites of CAR transduced Nalm-6 cells were analyzed by linear-amplification mediated PCR (LAM-PCR) and visualized with Circos plots. The integration sites across the genome and genomic features were shown from outer to inner circle: (1) cytogenetic bands; (2) genes that harbor these integration sites along with a bar chart showing the reads of integration sites; (3) the distribution of integration sites, with colored circles representing different gene functional regions of the host sequence: purple for promoter region, green for intron region, and red for distal intergenic region. **E** Phenotype changes of Nalm-6 cells transduced with small amount of CD19 CAR lentiviruses detected by flow cytometry over time. Gating was based on the same cells stained with isotype-matched antibody. **F** Dynamics of CD19^−^ B phenotype in relapsed cells after co-culture with different ratios (5×, 20×) of Nalm-6 cells. Gating was based on the same cells stained with isotype-matched antibody. **G** Relapsed CD19^−^ Nalm-6 cells were tested by qPCR specific for VSV-G sequence. **H** Comparison of in vitro efficacy of CD19-, CD22-, CD19/CD22- and CD22×CD19- CAR T cells. Cocultures with the relapsed cells were performed at 1:5, 1:1, and 5:1 E: T ratios, and lysis efficacies were detected by the LDH release assay **Declarations**

To further validate this hypothesis, we performed additional analysis on the relapsed cells and explored salvage therapies. We designed CAR sequence-specific primers (spanning 41BB-CD3ζ), along with probes, and carried out qRT-PCR, FCM, and confocal microscopy to demonstrate the expression of CAR in the relapsed CD19^-^ Nalm-6 cells. qRT-PCR analysis of FMC63, CD247 (CD3ζ), and 4-1BB genes and flow cytometry for CAR in relapsed cells showed higher expression levels of CAR structural components compared to those in Nalm-6 cells (Fig. 5A, B). Confocal microscopy also demonstrated the colocalization of CAR on the cell surface of the relapsed leukemia (Fig. 5C). Lentiviral integration site analysis of CD19^−^ Nalm-6 cells revealed 64 highly credible integration events. None of the integration sites were found in the exon region, while two integration sites were located in the intron region of the proto-oncogenes/tumor suppressor genes (GNB1, PGBD5). Additionally, 10 sites were located within 10 Kb upstream of the transcription start sites (TSS), and 11 integration sites were located within 10 Kb near CpG islands (Fig. 5D). Then, we used a small amount of CD19 CAR lentivirus (M.O.I. = 0.2) to transduce Nalm-6 cells in order to simulate the preparation of CAR19-Nalm-6 cells. Nalm-6 cells showed a dramatic decrease in CD19 expression as observed via flow cytometry after 3 days, and only a small proportion of CD19^+^ Nalm-6 cells were detectable after 13 days (Fig. 5E). This result demonstrated that the transduction and expression of CD19 CAR in some Nalm-6 cells led to the loss of CD19 detection throughout the cell population. For potential replication-competent lentivirus (RCL), three relapsed CD19^−^ Nalm-6 lots were tested by qPCR specific for envelope sequences (residual VSV-G transfer to cells), and the results were negative (Fig. 5G). Then, we co-cultured the relapsed cells with Nalm-6 cells at different ratios (1:5 and 1:20). Although the percentage of CD19^−^ cells initially increased, there was no observed loss of CD19 antigen in Nalm-6 cells after 14 days of co-culture (Fig. 5F).

As patients with CD19-negative relapsed leukemia have a very poor prognosis, there is an urgent need to find safe and effective alternative treatments. Anti-CD22 CAR-T cells have been proven to effectively mediate anti-leukemic effects. We therefore constructed CART-22 and tandem CART-22 × 19 cells to analyze their therapeutic effects on relapsed CD19^−^ Nalm-6 cells (Fig. 5H; Additional file 4: Fig. 3). The cytotoxic activity of the CART-19 cells against Nalm-6 cells was higher than that of the relapsed cells at 18 h. At 24 h, CART-22 cells demonstrated equally strong anti-tumor effects on Nalm-6 cells and the relapsed cells. Their cytotoxicity against the relapsed cells was higher than that of CART-19 and CART-19/22 cells at the E: T ratio of 5:1. Meanwhile, the killing activity of tandem CART-22 × 19 cells against the relapsed cells at an E: T ratio of 5:1 was consistent with the effect of CART-22, both achieving more than 80% efficacy. Thus, CART-22 and tandem CAR-T cell therapy could be used as rescue or combination therapy for leukemia cells that have lost the CD19 epitope induced by CAR transduction.

## Discussion

To date, both preclinical and clinical trials of anti-CD19 CAR-T cells have produced promising results. There are currently two approved CAR-T products for patients with r/r B-ALL: tisagenlecleucel (CTL109, Kymriah©) has been validated for use in children and young adults up to the age of 25, while brexucabtagene autoleucel (Tecartus©) is approved for adult patients. In the tisagenlecleucel phase II and IIb clinical trials (the ELIANA trial, NCT02435849), the complete response (CR) rate was 83%, with the relapse and CD19-negative relapse rates of 30% and 67%, respectively [24, 25]. Since 2017, real-world data gathered from patients receiving commercial Kymriah treatment has indicated CR rates ranging from 64 to 96%, with relapse rates ranging from 22 to 48%. Of these, CD19 negative relapse rates range from 33% ∼ 50% [25–28]. Other CAR T cells with FMC63-derived CD19-specific scFv have been evaluated in r/r B-ALL patients in several clinical trials. Among these trials, the relapse rates after anti-CD19 CAR-T cell therapy range from 22 to 53%, while the relapse rates of CD19-negative cases range from 30 to 75% [29–32]. Overall, most B-ALL patients (62% to 96%) achieved CR after anti-CD19 CAR-T therapy, although relapses were observed in a consistent percentage of patients (ranging from 22 to 53%) who initially achieved remission. So, relapse is the main reason for the failure of this otherwise highly successful therapy, wherein CD19-negative relapses carry a worse prognosis compared to CD19-positive relapses and create a significant challenge for potential follow-up treatments [28].

CD19 is a key molecule in the maturation and differentiation of B lymphocytes and has an important role in B-cell receptor signaling [33, 34]. Its loss is a dominant form of B-ALL relapse after CART-19 cell therapy, as mentioned above. Studies have suggested various biological and molecular mechanisms that contribute to the emergence of CD19-negative leukemic blasts after CAR-T therapy, including genetic mutations, alternative splicing, lineage switching, epitope masking, decreased CD19 antigen density, and trogocytosis [10]. However, the incidence rate of these mechanisms is not completely clear in clinical practice. Our results from the in vitro cell model system showed that Ag-negative blasts were obtained after a cytotoxicity assay without applying CRISPR technology, which may involve multiple resistance mechanisms.

Current strategies used to prevent CD19-negative relapse include the application of CAR-T cell therapy with two or more antigen-specific CAR-T cells, usually CD19 and CD22, as well as fourth-generation CAR-T cells. Dual-targeted CD19/CD22 CAR-T cells do not completely prevent CD19-negative relapse, as evidenced by reported cases in published studies. As for fourth-generation CAR-T cells, there is insufficient clinical data to draw conclusions. Similarly, the available data on specific strategies for the mechanism of CD19-negative relapse is very limited. In this sense, finding a model suitable for studying CD19-negative r/r B-ALL in vitro is critical to address the most important problem in this patient population.

Given the availability of the anti-CD19 scFv, FMC63, and recent clinical advances in CART-19 therapy, we sought to first explore the efficacy and optimal configuration of GMP-grade CART-19 cells against B-ALL. We selected the CD19-BBζ CAR for further testing and T cells were efficiently transduced with lentiviruses encoding CD19-BBζ. We demonstrated that genetically engineered T cells expressing CD19-BBζ CAR could be successfully expanded with low-serum medium, and half of these cells have the phenotype of less differentiated T cell subsets. Our results suggested that CART-19 cells possess rapid tumor recognition ability and display enhanced antileukemic activities, such as cytolytic capacity and cytokine production, at low E: T ratios compared to mock T cells in vitro. Then, we used NSG mice to evaluate the therapeutic effectiveness of CART-19 cells in vivo. The persistence and proliferation of infused CAR-T cells in the circulation have been shown to be critical for tumor eradication following adoptive CAR-T cell infusion. Our results indicated that CART-19 cells exhibit durable enhanced anti-tumor activity without obvious toxicities in animal model.

In the in vitro cytotoxicity assay described above, co-cultured cells showed no viable Nalm-6 cells after prolonged incubation. Furthermore, CD19-positive blasts were detected in the bone marrow of a dying mouse by flow cytometry on day 42 of the in vivo study. As such, we did not observe CD19-negative relapses. Then, we shortened the preparation time of CAR-T cells and cultured them under serum-free culture conditions, following cytotoxicity analysis of the CAR-T cells two days after transduction. Surprisingly, we did see differences in cytolytic activity at low E: T ratios of 1:5 and 1:1. On day 7, we identified the presence of CD10^+^CD19^−^ cells in the co-cultured cells, and these clones were cultured and proliferated for an extended period while still maintaining the CD19-negative phenotype. CD19-negative relapses revealed consistent growth characteristics with Nalm-6 cells, including doubling time, cell cycle, and karyotype. However, the expression of *CD19* and *PAX5* in relapses decreased by half compared to Nalm-6 cells. A single relapse cell was selected and cultured via limiting dilution assays, and we confirmed the relapsed subclones expressed less and smaller CD19 protein whose intracellular domains could be identified. Moreover, the expression level of CD81 on the cell surface was normal, while a small amount of the CD19 extracellular domain was detected in the cytoplasm around the nucleus. In addition, we excluded other possible mechanisms of Ag-negativity, including pre-existing CD19-negative Nalm-6 cells (Additional file 5: Fig. 4), CD19 splicing variants, structural alterations of the B cell receptor complex, and promoter DNA hypermethylation. Theoretically, a down-regulation that reduces the number of CD19 molecules on the membrane surface is reversible in the absence of CART-19 cells and genetic mechanisms leading to a total loss of the CD19 antigen, indicating additional escape mechanisms for CD19-negative relapse in this study.

Through RNA-seq results, we found that the CD247 and CD8A genes were significantly upregulated in the relapsed cells and subsequently confirmed that the relapsed Nalm-6 cells have been transduced with the FMC63-4-1BB-CD3ζ lentiviral vector. We suspected that the CAR-positive relapses were generated through lentiviral transduction, which occurred either via RCL or during the co-culture process of the cytotoxicity assay. We successfully repeated this Ag-negative phenomenon in Nalm-6 cells transduced with microamounts of CAR19 lentivirus, but these cells deteriorated after 10 generations in culture. We then ruled out RCL as a cause by TaqMan qPCR and co-culturing the relapsed cells with the original Nalm-6 cells without finding CD19 antigen loss. We therefore hypothesized that lentiviral particles adhering to the surface of CART-19 cells prepared in a short period of 3 days infected Nalm-6 cells during co-culture, and then persistent CAR expression as well as the down-regulation and truncation of CD19 expression induced Ag-negative relapses. Moreover, CART-22 cells can effectively kill these relapsed cells but cannot eradicate them either. Besides general strategies of multitargeting or target independent lysis, specific approaches should be developed to address the mechanisms of CD19 antigen loss after CART-19 therapy. The target negative cell line in this study is ideal for research purposes and has the potential to be used to create animal models of r/r B-ALL.

Several drawbacks should be taken into account when interpreting the findings of this study. First, we used immunofluorescence and western blotting techniques to identify the presence and localization of CD19 in relapsed cells, without conducting direct sequencing of CD19 molecules or detecting post-translational modifications. Additional research is required to analyze and engineer the CD19-negative Nalm-6 cells. Second, as CAR transduction into a single tumor cell has been reported [35], current advancements in manufacturing technologies have applied a closed automated manufacturing system and used microbeads for magnetic enrichment to separate T cells without the issue of residual tumor blasts contamination. Consequently, the occurrence of CAR transduction into tumors has been eliminated in clinical applications. Moreover, novel strategies need to be developed to tackle resistance mechanisms following CART-19 therapy.

In conclusion, we have successfully generated CAR-T cells that specifically target CD19 and obtained a CD19-negative refractory relapsed B-ALL cell line, providing a new in vitro model for exploring the treatment of r/r B-ALL patients with low antigen density.

### Electronic supplementary material

Below is the link to the electronic supplementary material.

**Additional file 1: Table 1.** Sets of primers used for PCR amplification and Real Time-qPCR amplification. **Table 2.** Clinical characteristics and sequencing of CD19 transcripts in B-ALL patients.

**Additional file 2: Fig. 1.** Morphology and immunophenotyping of Nalm-6 cells co-cultured with CART-19 cells for approximately 27 days. The CART-19 cells were expanded in the TexMACS Medium upplemented with IL-2 and 5% FBS.

**Additional file 3: Fig. 2.** Growth characteristics of relapsed CD19-negative Nalm-6 cells. **A** Representative cell cycle profiles of Nalm-6 cells and relapsed CD19- Nalm-6 cells (*n* = 3). **B** Karyotype analysis of Nalm-6 cells and relapsed CD19^−^ Nalm-6 cells. The karyotypes of them were described as 46, XY, del (5)(q22q35) (2)/46, XY (16) and 46, XY, del (5)(q22q35) (8)/46, XY (12), respectively.

**Additional file 4: Fig. 3.** Expansion and phenotypic identification of anti-CD22 CAR-T cells. **A** Schematic of CD19-CAR, CD22-CAR and CD22 × 19 bivalent-CAR structure. **B** Cell amplification curve. **C** CAR-T cell phenotypic characteristics. **D** Surface expression of CD19-CAR, CD22-CAR and CD22 × 19-CAR on T cells were analyzed by flow cytometric histograms.

**Additional file 5: Fig. 4.** Sorting, expansion and phenotypic identification of CD10^+^CD19^−^ cells from Nalm-6 cells. The phenotypic characteristics of cells were analyzed by flow cytometry on day 0, day 11, and day 22.

## Data Availability

The datasets supporting the findings of this study are available from the corresponding author on reasonable request.

## Abbreviations

ALL: Acute lymphoblastic leukemia
r/r B-ALL: Relapsed/refractory B lineage acute lymphoblastic leukemia
allo-HSCT: Allogeneic hematopoietic stem cell transplantation
CAR-T: Chimeric antigen receptor T cell
CART-19: Anti-CD19 CAR-T
scFv: Single-chain variable fragment
OS: Overall survival
DFS: Disease-free survival
E: T ratio: Effector: Target (E: T) ratio
MFI: Mean fluorescence intensity
WT: Wild-type
TSS: Transcription start site
