# Supplementary material for: Unraveling resistance mechanisms in anti-CD19 chimeric antigen receptor-T therapy for B-ALL: a novel in vitro model and insights into target antigen dynamics
Source: J Transl Med. 2024 May 21;22:482. doi: 10.1186/s12967-024-05254-z (PMC11110321; doi:10.1186/s12967-024-05254-z)
Supplement: Supplementary file 1 — Additional file 1: Table 1. Sets of primers used for PCR amplification and Real Time-qPCR amplification. Table 2. Clinical characteristics and sequencing of CD19 transcripts in B-ALL patients. [file 12967_2024_5254_MOESM1_ESM.docx]

# Supplementary Information

**Table 1.** Sets of primers used for PCR amplification and Real Time-qPCR amplification

| Target | Direction | Sequence (5' to 3') |
| --- | --- | --- |
| 4-1BB | Fwd | TGCCGATTTCCAGAAGAAGAAGAAG |
|  | Rev | GCGCTCCTGCTGAACTTC |
|  | Probe | ACTCTCAGTTCACATCCTC |
| VSV-G | Fwd | TCAAAGGCTCAGGTGTTCGA |
|  | Rev | CATCAGGAAGTTGCGAAGCA |
|  | Probe | CATCCTCACATTCAAGACG |
| CD3ζ | Fwd | GATTTCCAGAAGAAGAAGAAGGAGGA |
|  | Rev | GGTTCTGGCCCTGCTGGTA |
|  | Probe | TGTGAACTGAGAGTGAAGT |
| FMC63 | Fwd | TCTGGGAGACAGAGTCACCAT |
|  | Rev | CACTGCCACTGAACCTTGATG |
| CD247 | Fwd | GCCGAGAAGGAAGAACCCTC |
|  | Rev | GTGGCTGTACTGAGACCCTG |
| PAX5 | Fwd | ACTTGCTCATCAAGGTGTCAG |
|  | Rev | TCCTCCAATTACCCCAGGCTT |
| Actin | Fwd | AGCATCCCCCAAAGTTCAC |
|  | Rev | AAGGGACTTCCTGTAACAACG |

**Table 2.** Clinical characteristics and sequencing of CD19 transcripts in B-ALL patients

| Patient number | 1 | 2 | 3 | 4 |
| --- | --- | --- | --- | --- |
| Age | 3y6m | 3y | 12y | 5y5m |
| Gender | Male | Male | Male | Male |
| Leukemia type | B-ALL (CD45^low^CD19^+^CD34^low^CD10^+^CD58^+^) | B-ALL (CD45^-^CD19^+^CD34^low^CD10^+^CD58^+^) | B-ALL (CD45^-^CD19^+^CD34^-^CD10^+^CD58^+^) | B-ALL (CD45^-^CD19^+^CD34^low^CD10^+^CD58^+^) |
| Transcript Variants |  |  |  |  |
| Number of sequencing samples | 12 | 9 | 14 | 11 |
| Transcript 1 or 2 (WT) | 2 | 5 | 2 | 2 |
| partΔExon2 (-130bp) | 3 | 2 | 1 | 1 |
| Intron2 | 5 | 2 | 3 | 3 |
| Intron2+Intron6 |  |  | 2 |  |
| partΔExon2+Intron2 | 1 |  | 1 | 1 |
| ΔExon6 |  |  |  | 1 |
| partΔExon2+Intron2+ΔExon6 |  |  |  | 1 |
| Intron2+ΔExon6 |  |  | 4 | 2 |
| Intron6 |  |  | 1 |  |
| partΔExon2+Intron2+ΔExon5+ΔExon6 | 1 |  |  |  |
