# Supplementary material for: Unraveling resistance mechanisms in anti-CD19 chimeric antigen receptor-T therapy for B-ALL: a novel in vitro model and insights into target antigen dynamics
Source: J Transl Med. 2024 May 21;22:482. doi: 10.1186/s12967-024-05254-z (PMC11110321; doi:10.1186/s12967-024-05254-z)
Supplement: Supplementary file 2 — Additional file 2: Fig. 1. Morphology and immunophenotyping of Nalm-6 cells co-cultured with CART-19 cells for approximately 27 days. The CART-19 cells were expanded in the TexMACS Medium upplemented with IL-2 and 5% FBS. [file 12967_2024_5254_MOESM2_ESM.docx]

# Supplementary Information


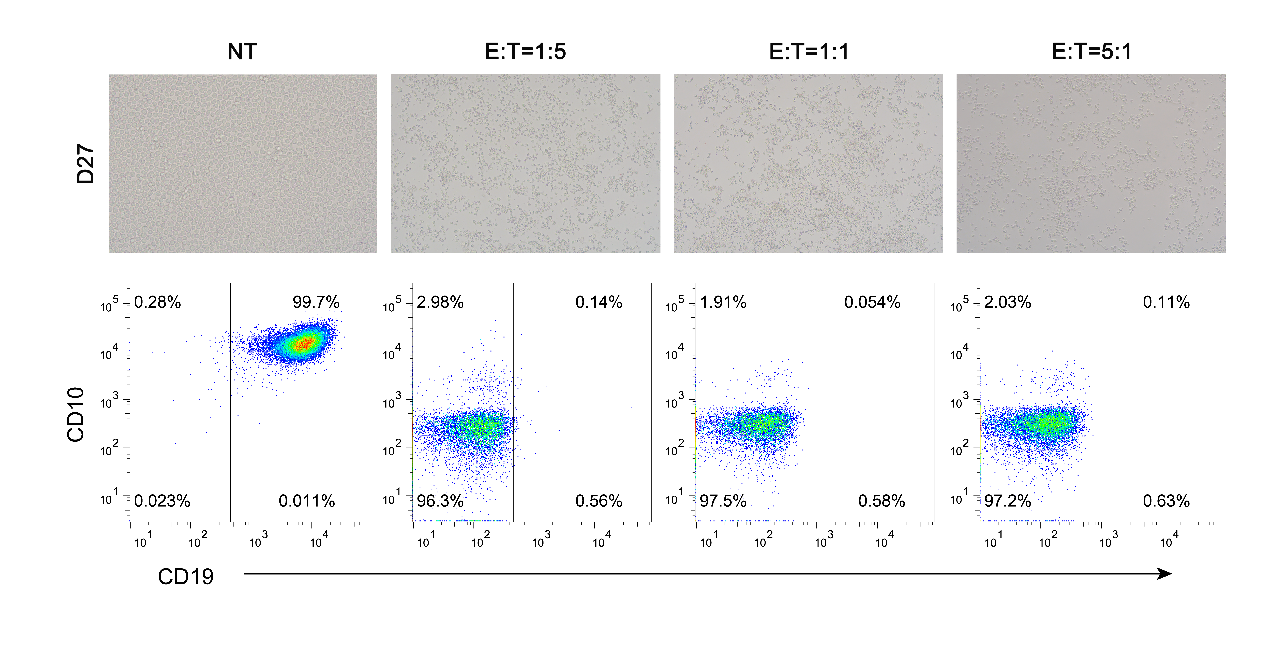


**Figure 1.** Morphology and immunophenotyping of Nalm-6 cells co-cultured with CART-19 cells for approximately 27 days. The CART-19 cells were expanded in the TexMACS Medium upplemented with IL-2 and 5% FBS.
