# Supplementary material for: Unraveling resistance mechanisms in anti-CD19 chimeric antigen receptor-T therapy for B-ALL: a novel in vitro model and insights into target antigen dynamics
Source: J Transl Med. 2024 May 21;22:482. doi: 10.1186/s12967-024-05254-z (PMC11110321; doi:10.1186/s12967-024-05254-z)
Supplement: Supplementary file 3 — Additional file 3: Fig. 2. Growth characteristics of relapsed CD19-negative Nalm-6 cells. A Representative cell cycle profiles of Nalm-6 cells and relapsed CD19- Nalm-6 cells (n = 3). B Karyotype analysis of Nalm-6 cells and relapsed CD19− Nalm-6 cells. The karyotypes of them were described as 46, XY, del (5)(q22q35) (2)/46, XY (16) and 46, XY, del (5)(q22q35) (8)/46, XY (12), respectively. [file 12967_2024_5254_MOESM3_ESM.docx]

# Supplementary Information


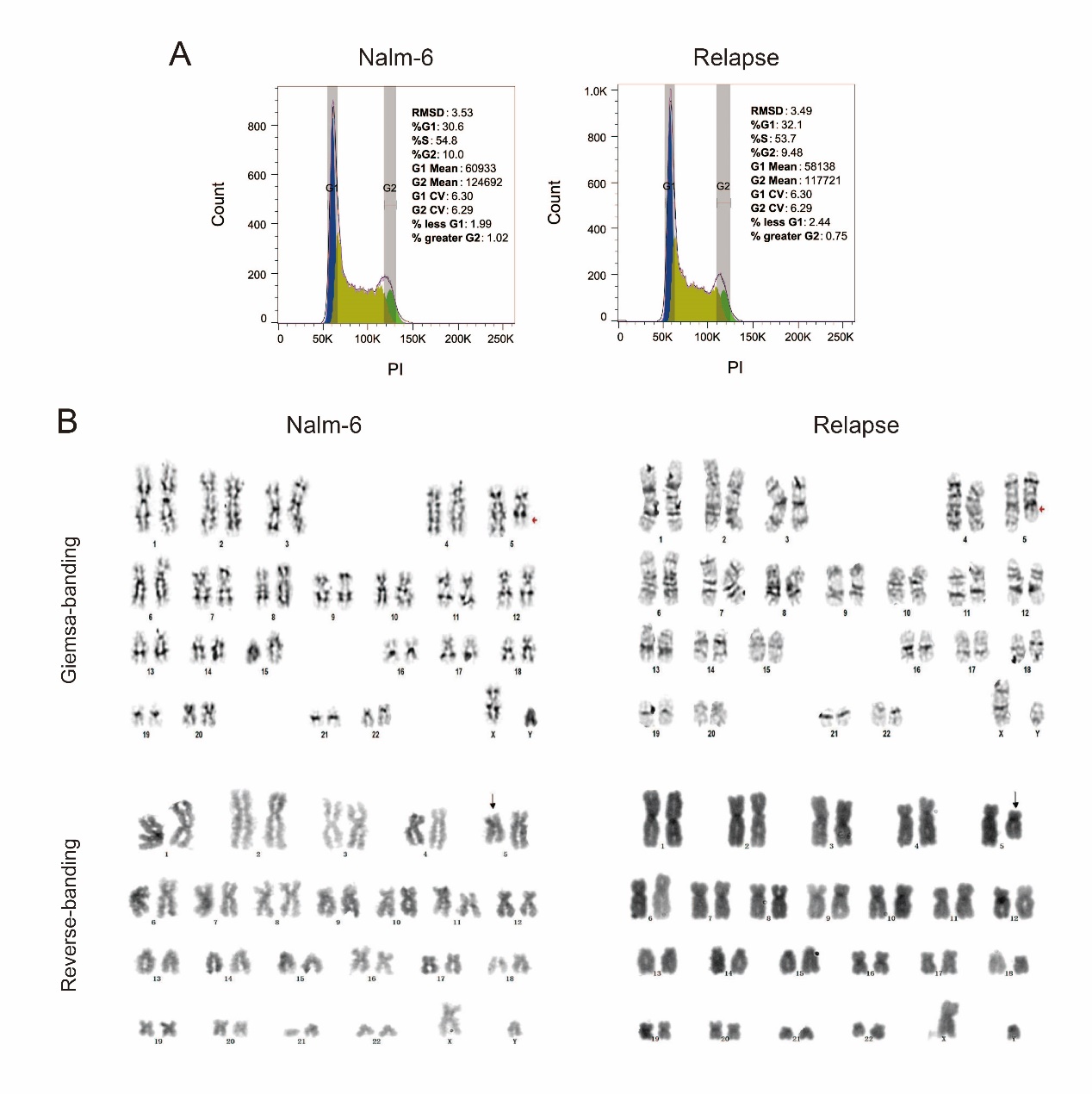


**Figure 2.** Growth characteristics of relapsed CD19-negative Nalm-6 cells. **A** Representative cell cycle profiles of Nalm-6 cells and relapsed CD19- Nalm-6 cells (n = 3). **B** Karyotype analysis of Nalm-6 cells and relapsed CD19^-^ Nalm-6 cells. The karyotypes of them were described as 46, XY, del (5)(q22q35)[2]/46, XY[16] and 46, XY, del (5)(q22q35)[8]/46, XY[12], respectively.
