# Supplementary material for: Unraveling resistance mechanisms in anti-CD19 chimeric antigen receptor-T therapy for B-ALL: a novel in vitro model and insights into target antigen dynamics
Source: J Transl Med. 2024 May 21;22:482. doi: 10.1186/s12967-024-05254-z (PMC11110321; doi:10.1186/s12967-024-05254-z)
Supplement: Supplementary file 4 — Additional file 4: Fig. 3. Expansion and phenotypic identification of anti-CD22 CAR-T cells. A Schematic of CD19-CAR, CD22-CAR and CD22 × 19 bivalent-CAR structure. B Cell amplification curve. C CAR-T cell phenotypic characteristics. D Surface expression of CD19-CAR, CD22-CAR and CD22 × 19-CAR on T cells were analyzed by flow cytometric histograms. [file 12967_2024_5254_MOESM4_ESM.docx]

# Supplementary Information


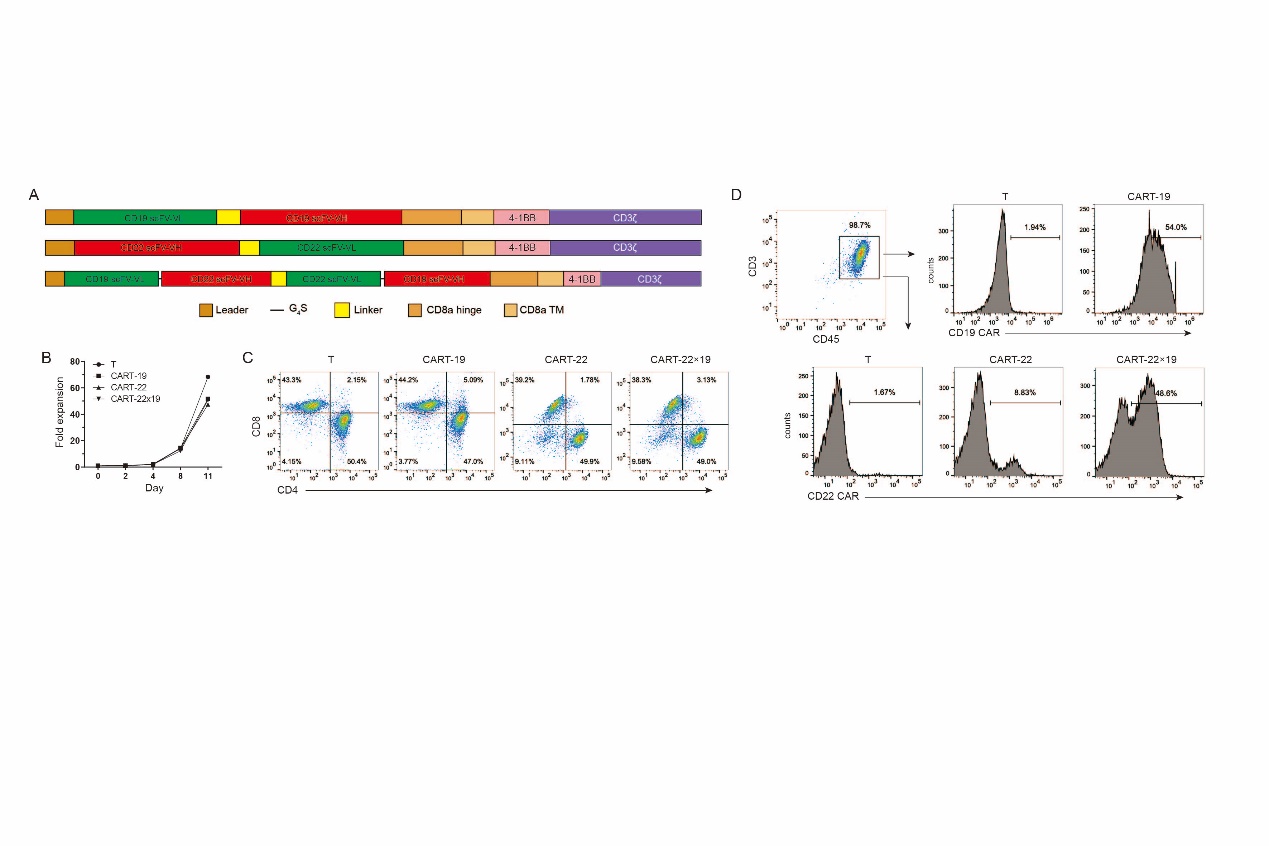


**Figure 3.** Expansion and phenotypic identification of anti-CD22 CAR-T cells. **A** Schematic of CD19-CAR, CD22-CAR and CD22×19 bivalent-CAR structure. **B** Cell amplification curve. **C** CAR-T cell phenotypic characteristics. **D** Surface expression of CD19-CAR, CD22-CAR and CD22×19-CAR on T cells were analyzed by flow cytometric histograms.
