# Supplementary material for: Unraveling resistance mechanisms in anti-CD19 chimeric antigen receptor-T therapy for B-ALL: a novel in vitro model and insights into target antigen dynamics
Source: J Transl Med. 2024 May 21;22:482. doi: 10.1186/s12967-024-05254-z (PMC11110321; doi:10.1186/s12967-024-05254-z)
Supplement: Supplementary file 5 — Additional file 5: Fig. 4. Sorting, expansion and phenotypic identification of CD10+CD19− cells from Nalm-6 cells. The phenotypic characteristics of cells were analyzed by flow cytometry on day 0, day 11, and day 22. [file 12967_2024_5254_MOESM5_ESM.docx]

# Supplementary Information


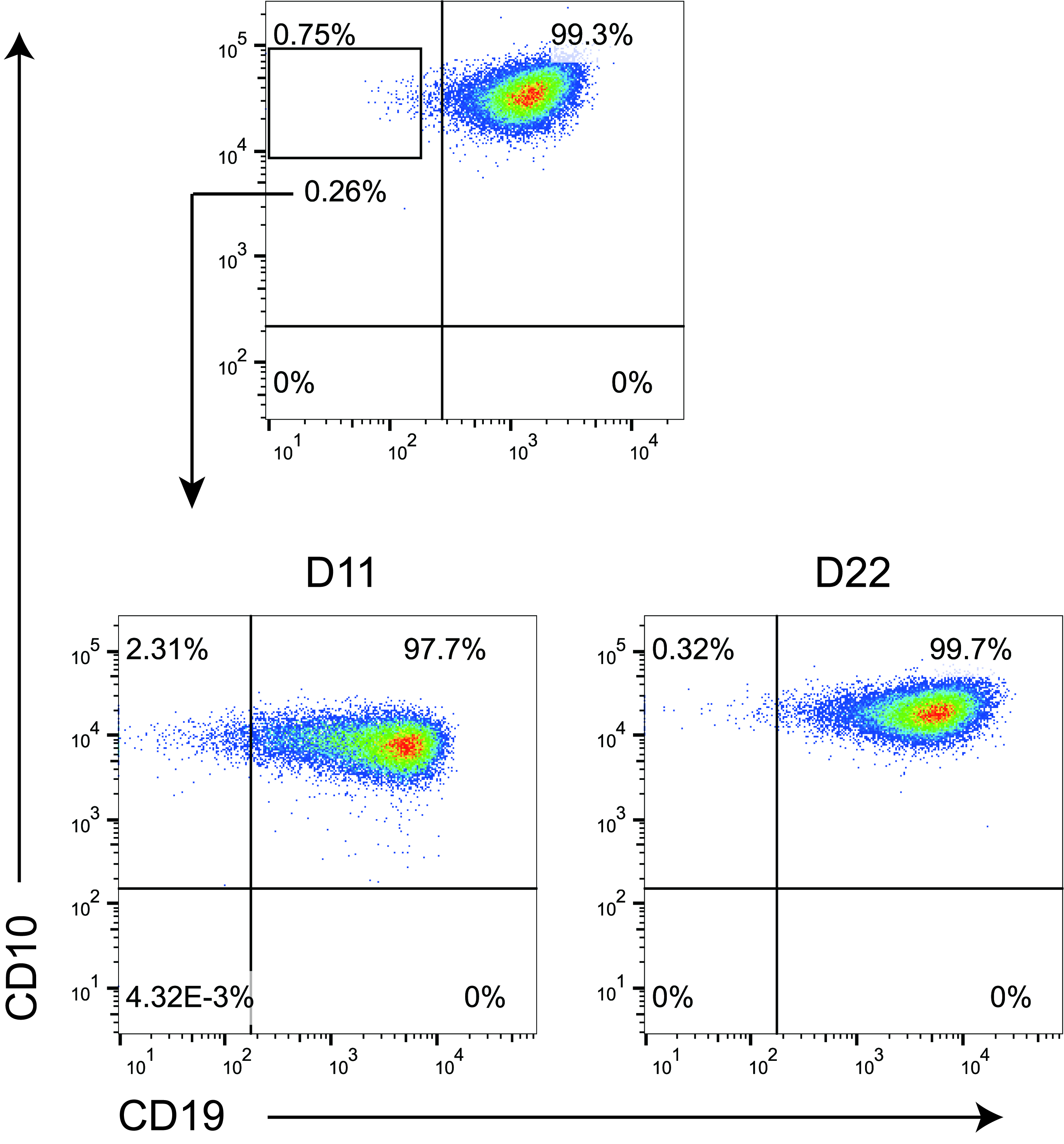


**Figure 4.** Sorting, expansion and phenotypic identification of CD10^+^CD19^-^ cells from Nalm-6 cells. The phenotypic characteristics of cells were analyzed by flow cytometric on day 0, day 11, and day 22.
